# Heat Stability and Icing Delay on Superhydrophobic Coatings in Facile One Step

**DOI:** 10.3390/polym14153124

**Published:** 2022-07-31

**Authors:** Jingyu Shang, Yongfeng Jiang, Wenhua Wang

**Affiliations:** 1 College of Mechanical and Electrical Engineering, Hohai University, Changzhou 213000, China; 2 Jiangsu Province Wind Power Structural Research Center, Nanjing 211100, China; 3 Quick Intelligent Equipment Co., Ltd., Changzhou 213000, China; wwhgigi@163.com

**Keywords:** superhydrophobic, stability, icing delay, MTMS, ZnO, SiO_2_

## Abstract

Superhydrophobic coatings are limited to poor durability and a tedious preparation process. In this work, an efficient, eco-friendly, and cost-effective sol-gel method is developed for preparing superhydrophobic surfaces using an all-in-one suspension composed of methyltrimethoxysilane (MTMS), nano silicon dioxide (SiO_2_) particles, and micron zinc oxide (ZnO) particles. Superhydrophobic coatings with a contact angle (CA) up to 153.9° and a sliding angle (SA) of about 3.0° are prepared on Q235 steel substrates using MTMS 5 mL, 0.8 g of nano SiO_2_, and 0.2 g of micron ZnO. The morphology of the superhydrophobic coating is characterized by scanning electron microscopy (SEM), and the surface is covered with a micro- and nano-scaled hierarchical rough structure. A series of tests are conducted, including long-term stability tests and thermostability tests. The CAs are all above 150°, and the SAs are below 6.3°, indicating the excellent static stability of the prepared superhydrophobic coatings. Moreover, the CA of the superhydrophobic coating remains above 152° after 120 h of UV exposure, and the time for a water droplet to freeze on the surface of the superhydrophobic coating is 18 times of the bare Q235 steel, indicating that the superhydrophobic coating exhibits good resistance to UV radiation and icing-delay properties.

## 1. Introduction

Superhydrophobicity is found on plant surfaces and the bodies of some insects, such as lotus leaves, butterfly wings, and the legs of water striders [1,2]. It was discovered that superhydrophobic properties are determined by the synergy of surface micro and nano rough structures and low surface energy material modifications [3,4]. A superhydrophobic coating is characterized by a water contact angle (WCA) over 150° and a sliding angle (SA) lower than 10° [5]. Superhydrophobic surfaces are widely used in exterior wall coatings [6], textiles [7,8], metal corrosion protection [9,10], anti-bacterial [11,12], anti-fogging, and self-cleaning [13,14,15]. Especially in the field of icephobic coating in harsh outdoor environments, superhydrophobic technology has a wide range of applications [16,17]. Therefore, the preparation of superhydrophobic surfaces has been investigated from several angles by academics and industry to enhance their process capabilities. At present, nearly 10 methods for the preparation of superhydrophobic surfaces have been developed, mainly etching [18,19], vapor deposition [20,21], hydrothermal method [22,23], electrostatic spinning [24,25], template method [26,27], sol-gel method [28,29], electrodeposition [30,31], self-assembly method [32,33], and plasma deposition [34,35].

Amongst these methods, sol-gel method is favored by researchers because of its mild reaction conditions and simple process. Silica sols were produced by ethyl orthosilicate catalyzed by ammonia and then modified by perfluorodecyltrimethoxysilane to produce superhydrophobic coatings [36]. Silica sols are produced via ethyl orthosilicate catalyzed by hydrochloric acid [37]. The silica sols were catalyzed by hydrochloric acid and ammonium hydroxide and subsequently modified with expensive fluorosilanes [38]. In the above methods of preparing superhydrophobic coatings, the preparation of sols requires 24 h of aging. It is time-consuming, and a wide variety of chemical reagents were used.

Superhydrophobic surfaces applied for icephobic membranes have been widely reported [16,39,40]. The superhydrophobic coatings prepared by methyl methacrylate, glycidyl methacrylate, heptadecafluorodecyl acrylate, and poly (vinylidene) fluoride also exhibited superior chemical stability, self-cleaning property, and icing-delaying ability [39]. A robust transparent superhydrophobic coating was developed using an alkoxysilane binder, fumed SiO_2_ nanoparticles, and methyltriethoxysilane (MTES). In addition, the superior icephobic behavior of the coating was confirmed [40]. An icephobic coating of a self-formed superhydrophobic surface using polydimethylsiloxane (PDMS) and SiO_2_ nanoparticles was investigated. Polyvinylidene fluoride (PVDF) was added to the PDMS solution to improve the mechanical properties of the icephobic layer. Moreover, the effect of superhydrophobicity on icephobicity was determined in different sizes of the SiO_2_ nanoparticles. Furthermore, the ice adhesion strength increases with the increase of the SiO_2_ nanoparticles powder size [16].

According to the published articles, superhydrophobic coatings of ZnO or MTMS have been widely reported. ZnO particles and molecular sieves are fused into PDMS films to prepare superhydrophobic coatings [41]. Branched hierarchical ZnO nanowires and ZnO nanowires were modified by MTMS, and the WCA of the coating was about 153 ± 3°. The coating showed the higher Cassie–Baxter stability [42]. Cui et al. analyzed the conditions and products of five siloxanes such as KH550, KH570, and MTMS for modification of silica sol. MTMS was finally selected as the modifier for silica sol because of its low experimental conditions and transparent products [43]. MTMS was hydrolyzed and mixed with silica sol at different parameters to form the hybrid nanocomposite coating [44]. Alkaline silica sols were modified by MTMS to prepare transparent hydrophobic coatings containing silicon with a low surface energy and high temperature resistance [45]. In conclusion, all the above scholars have successfully prepared superhydrophobic coatings. However, the operation process is tedious and complicated, and at least five kinds of chemical reagents were used in each preparation process.

To address the common problems of tedious preparation process, a superhydrophobic film based on MTMS, SiO_2_ nanoparticles, and micron ZnO particles by facile sol-gel was prepared. The preparation of silicone dioxide sols took only 1 h. The superhydrophobic coating was successfully produced using only three chemical reagents. In this paper, a superhydrophobic surface with a two-level bionic rough structure using SiO_2_ nano powder combined with micron ZnO is innovatively presented. It is worth mentioning that the superhydrophobic films have good ultraviolet (UV) resistance, icing-delay capacity, film formation, self-cleaning characteristics, and environmental friendliness.

## 2. Materials and Methods

### 2.1. Reagents and Materials

The size of Q235 steel substrate was 20 mm × 20 mm × 1 mm. Hydrofluoric acid (HF, 0.4 mol/L, 25 °C) was purchased from Nantong Mingxin Chemical Co., LTD (Nantong, China). Methyltrimethoxysilane (MTMS, purity ≥ 98.0%, 25 °C) was purchased from Runhui Biotechnology Co., LTD (Weihai, China). The SiO_2_ nanoparticles with specific surface area of 220 ± 30$\;m^{2}/g$ and diameter of 30 ± 5 nm were purchased from Shanghai Kaiyin Chemical Co., LTD (Shanghai, China). Zinc oxide (ZnO, particle size 25 μm, analytical purity) was purchased from Tianjin Hengxing Fine Chemical Co., LTD (Tianjin, China). All chemical and reagents were used as received without further treatment. The water used in all experiments and tests was obtained from the UPC-III water purification system.

### 2.2. Preparation

#### 2.2.1. Q235 Steel Substrate Pretreatment

**Figure 1:**
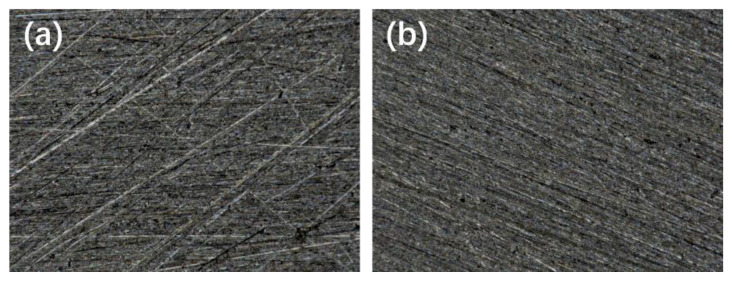
(**a**) Q235 steel sanded with 500# sandpaper and (**b**) Q235 steel activated with hydrofluoric acid for 1 min.

The surface of the Q235 steel was sanded with 500# sandpaper and wiped clean with alcohol wool. Subsequently, the substrate was activated with hydrofluoric acid for 1 min, cleaned with deionized water, and dried. The pretreatment of Q235 steel substrates was carried out at room temperature (25 °C). Microscopic images of the two substrates are shown in Figure 1. The microscope images were taken using Keyence VHX-2000 digital Microscope. The magnification is 200×. After activation treatment with hydrofluoric acid, the surface texture was uniform and fine. In contrast, it appeared that the surface of the substrate after sandpaper sanding only had many obvious scratches. The Q235 steel substrate was activated to reveal the active metal interface, which facilitates the improvement of the bond between the plating and the substrate.

#### 2.2.2. Different Amount of SiO_2_ and Modification Time

Five milliliters of MTMS was added separately to 10–20 mL beakers, numbered 1–10. Then, from 0.1 g to 1 g of SiO_2_ nanoparticles was added into beakers and stirred for 1 h at room temperature (22 °C). Five milliliters of MTMS was added separately to 10–20 mL beakers, numbered 11–20. Then, from 0.1 g to 1 g of SiO_2_ nanoparticles was added into beakers and stirred for 2 h at room temperature (22 °C). Five milliliters of MTMS was added separately to 10–20 mL beakers, numbered 21–30. Then, from 0.1 g to 1 g of SiO_2_ nanoparticles was added into beakers and stirred for 3 h at room temperature (22 °C). Then the stirred solution was applied dropwise to the Q235 substrate and left to dry at room temperature for 24 h. Then, the coatings Nos. 1–30 were heated at 330 °C in a chamber furnace for 30 min.

#### 2.2.3. Different Amount of ZnO

Five milliliters of MTMS was added separately to 7–20 mL beakers, numbered 31–37. Then, ZnO from 0.02 g to 0.5 g was added and stirred magnetically at room temperature (22 °C) for 2 h. The stirred solution was applied dropwise to the Q235 substrate and left to dry at room temperature for 24 h. It was found that the surface of No. 31–35 was flat, and the surface of Nos. 36 and 37 showed obvious cracks. Then, the coatings of Nos. 31–35 were heated in a chamber furnace at 330 °C for 30 min.

#### 2.2.4. Different Amount of SiO_2_ and ZnO

Five milliliters of MTMS was added separately to 5–20 mL beakers. Then 0.8 g of SiO_2_ nanoparticles was added, respectively, and stirred magnetically at room temperature for 1 h. Then 0.02 g, 0.05 g, 0.1 g, 0.2 g, and 0.3 g of micron-grade ZnO was added, respectively, and stirred magnetically at room temperature for 1 h, numbered 38–42. The uniform solution was applied dropwise to Q235 steel substrate and left to dry at room temperature for 24 h; then, the coating was heated in a chamber furnace at 330 °C for 30 min.

#### 2.2.5. Heating Temperature

The coatings were prepared with 5 mL MTMS, 0.8 g of SiO_2_ nanoparticles, and 0.2 g micron ZnO. To further verify the effect of temperature on coating wettability, the coatings made from the above formulations were tested for CA after treatment at room temperature, 130 °C, 230 °C, 330 °C, and 430 °C for 30 min, Nos. 43–47.

**Figure 2:**
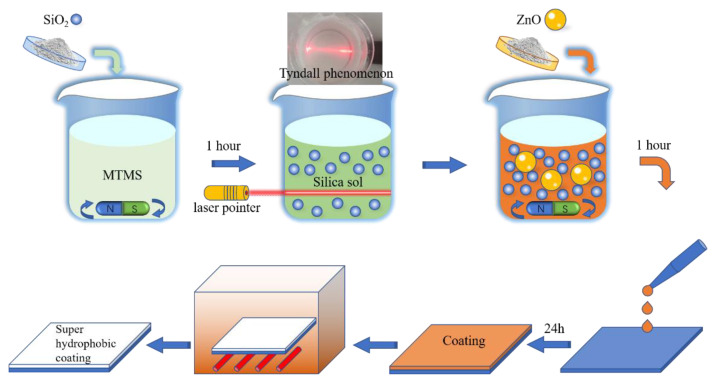
Diagram of coating preparation process.

The preparation process of the superhydrophobic coating is shown in Figure 2.

### 2.3. Characterization

Wettability of the coating was evaluated by a commercial CA instrument (Attension Theta by Biolin Scientific, Sweden). The test droplets were 4 μL distilled water droplets. The test sample was fixed on the sample table, a drop was placed on the sample table, the sample table was automatically tilted, the drop rolled down, and then the value of SA was recorded. Three random locations were selected at room temperature on the coating surface. The fitting method was Laplace fitting. The superhydrophobic surface morphology of the coating surface was characterized by scanning electron microscopy (SEM, JSM-IT100, Japan). A digital camera (Canon EOS 90D, Japan) was used to photograph the self-cleaning process. Two different particle sizes of contaminants (sand 150 μm and chalk powder 10 μm) were spread evenly on the superhydrophobic surface, and water droplets (0.25 mL) were used to clean the surface to evaluate the self-cleaning performance. The FTIR spectra were tested by Fourier transform infrared spectrometer (Tensor 27, Bruker, Germany) with the single reflection method in the range of 500–4000 cm^−1^. The resolution was 2 cm^−1^, and the samples were prepared by KBr compression method. The coatings were stored in air for 6 months to test their long-term stability. The thermal stability of the superhydrophobic coating was tested by two methods. The first was to heat the coating in a chamber furnace at 300 °C for different times. The second was to heat the coating in a chamber furnace at different temperatures for 30 min. Superhydrophobic coating heat treatment used a chamber furnace (Bo yuntong KF 1100). In addition, the bond strength between the coating and the Q235 steel substrate was evaluated by a tape peeling test; 3M 810 tape was used in the tape peeling test on the superhydrophobic coating. A sand impact test was conducted. Moreover, UV irradiation resistance and icing-delay tests of superhydrophobic coatings were conducted. The wavelength of the UV lamp was UVC-253.7, power 39W (Bobo home).

## 3. Results and Discussion

### 3.1. Processing

**Figure 3:**
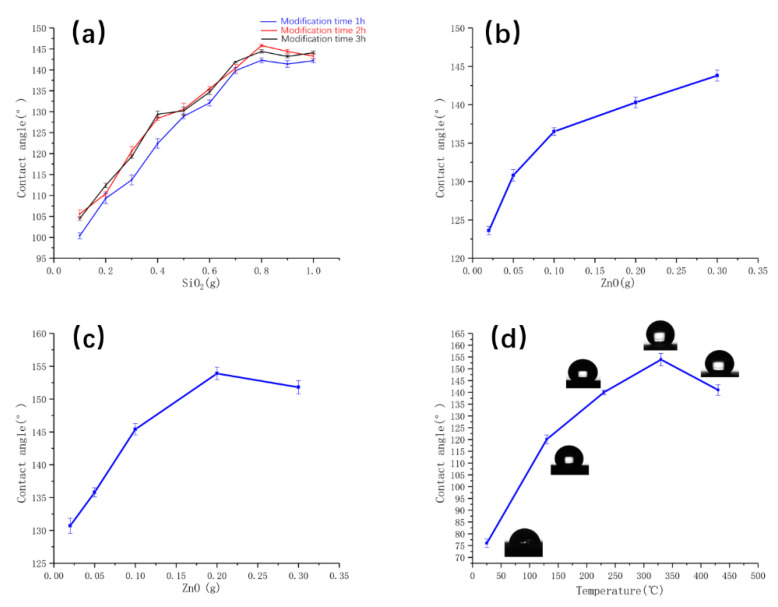
(**a**) CAs at different modification times and different SiO_2_ additions, (**b**) CA under different ZnO addition conditions, (**c**) Silicon dioxide 0.8 g, CA for different ZnO addition conditions, and (**d**) CAs of coatings at different temperatures.

The CAs of coatings 1–30 are shown in Figure 3a. The change of CA is shown in Figure 3. The results manifested that CA increased gradually with the increase of nano SiO_2_ from 0.1 g to 0.8 g. The CA decreased slightly, with the additional amount increasing to 1 g. The CA of the modification time of 2 h was longer than the modification time of 1 h with the same amount of nano SiO_2_. However, the CA was to some extent decreased, with the modification time increasing to 3 h. Therefore, the optimal test parameters were as follows, MTMS 5 mL, nano SiO_2_ 0.8 g, stirring time 2 h, static drying 24 h at room temperature, and heating temperature 330 °C.

With increasing SiO_2_ nanoparticles content, the WCA of the composite coating improved gradually. It was analyzed that when the addition of silica is low, the gap between the surface silica nanoparticles is large and cannot form a continuous support structure, so the surface water contact angle is small. When the amount of silica exceeded 0.8 g, the nano-silica tended to aggregate and form a cluster structure, making the surface structure gap smaller and the contact angle smaller.

The CAs of coatings 31–35 are shown in Figure 3b. Results manifested that CA increased from 123.6° to 143.8° with the increase of micron ZnO from 0.02 g to 0.3 g. With increasing ZnO micron particles content, the WCA of the composite coating improved gradually. It was analyzed that with the increase of zinc oxide addition, the surface formed a single-level rough structure with a certain structural gap.

The CAs were below 150° in the above two experimental groups. MTMS and nano SiO_2_ were used in the first set of experiments. MTMS and micron ZnO were used in the second set of experiments. Only one hierarchical particle of micron or nanometer was contained in the hydrophobic coatings prepared by the above two methods. The possibility of constructing micro and nano rough graded structures was explored to enable the coatings to achieve a superhydrophobic state in the following studies using micron-scale ZnO and nanoscale SiO_2_.

The CAs of coatings 38–42 are shown in Figure 3c. The results manifested that CA increased from 130.7° to a maximum of 153.9° with the increase of micron ZnO from 0.02 g to 0.2 g. However, the CA decreased to 151.8°, with the addition amount of ZnO increasing to 0.3 g. The superhydrophobic coating can be successfully produced using MTMS, micron ZnO, and nano SiO_2_. With increasing ZnO micron particles content, the WCA of the composite coating improved gradually. The contact angle reached a maximum value of 153.9° when zinc oxide was added at 0.2 g. It was analyzed that with the increase addition of ZnO, micro-nano hierarchical rough structures are formed on the surface. The optimal mass ratio of micron ZnO and nano SiO_2_ is 1:4.

CAs of coatings at different temperatures are shown in Figure 3d. The CA of the coating at room temperature was only 76.2°. The CA of the coating surface increased from 121° to 153.9° with the temperature increasing from 130 °C to 330 °C. The CA of the coating decreased to 141° after heating at 430 °C. The superhydrophobic coatings were obtained with 5 mL MTMS, 0.8 g of SiO_2_ nanoparticles, and 0.2 g micron-grade ZnO after heated in a chamber furnace at 330 °C for 30 min. In the following, the specimens tested and characterized were prepared using this process parameter, if not otherwise stated. Micron ZnO and nano-SiO_2_ built rough graded structures, and MTMS provided low surface energy.

### 3.2. Superhydrophobicity and Morphology

**Figure 4:**
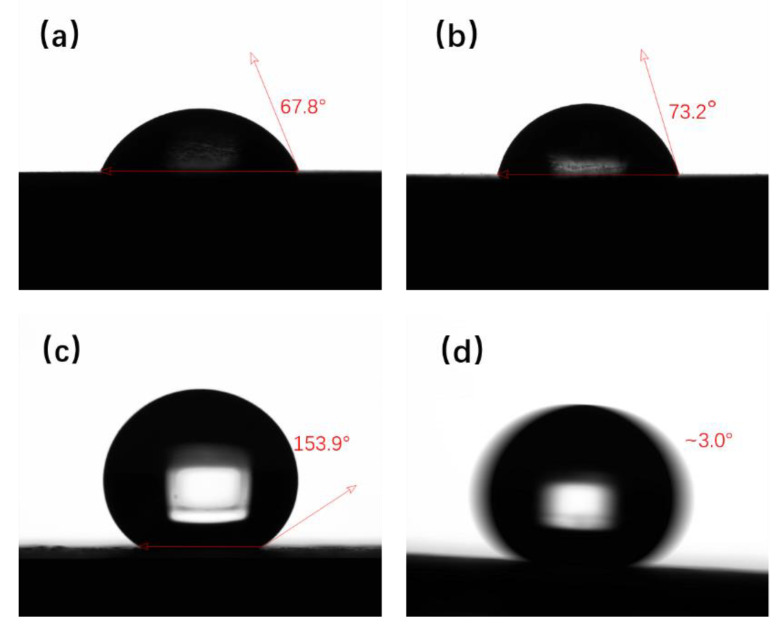
(**a**) CA of Q235 steel before grinding, (**b**) CA of Q235 steel after grinding by 500# sandpaper, (**c**) CA of superhydrophobic coating surface, and (**d**) SA of superhydrophobic coating surface.

In order to compare the Q235 steel substrate with the superhydrophobic coating, the contact angle was tested separately. The test results were shown in Figure 4. Q235 steel is a hydrophilic material with CAs of 67.8° and 73.2° before and after sanding by 500# sandpaper. The wettability of Q235 steel substrate is presented in Figure 4a,b. The superhydrophobic behavior with a water CA of 153.9° and SA of about 3.0° is shown in Figure 4c,d.

**Figure 5:**
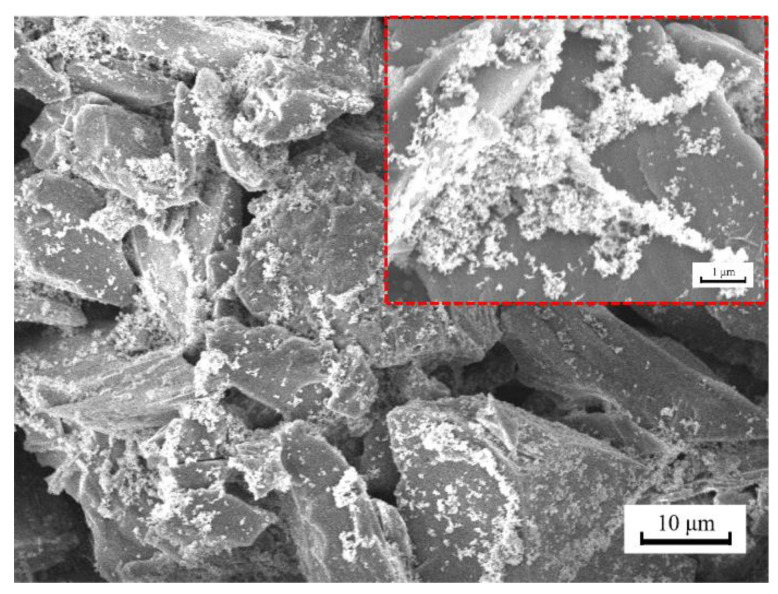
SEM image of superhydrophobic coating surface.

The surface morphology of the superhydrophobic coating is shown in Figure 5. The superhydrophobic surface was composed of rough, complex, accumulated micro and nano-block particles. It is the inhomogeneous rough structure and the low surface energy modification of the MTMS that constituted the superhydrophobic surface. The accumulation of the micro and nano particles formed rough hierarchical micro-nano structures. The rougher the surface, the smaller the liquid-solid contact surface and the more effective the hydrophobic effect. Apparently, there is a large space to trap air between these micro and nano particles. As a result, the actual contact area between the solid surface and the water droplet will be reduced, making the surface of Q235 steel significantly more hydrophobic [46]. The superhydrophobic surface has a hierarchical structure, in which ZnO resembles the protruding nubs in a lotus leaf as a primary structure, and the SiO_2_ nanoparticles attached to it can resemble the epidermal wax crystals in a lotus leaf. It was considered that the combination of the two-level hierarchical structures on the surface of SiO_2_-ZnO-MTMS stacked film, like the surface of lotus leaves, and the low surface energy MTMS substrate was the root cause of their superhydrophobicity [41].

**Figure 6:**
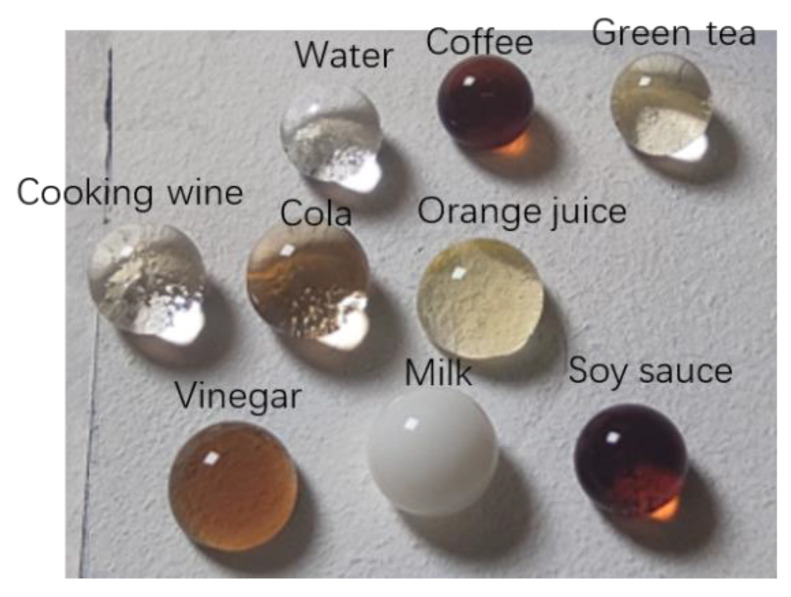
Morphology diagram and CA of different liquids on superhydrophobic surface, morphology diagram.

The shapes of water, milk, green tea, vinegar, cola, wine, soy sauce, orange juice, and coffee on the surface of superhydrophobic coating are shown in Figure 6. It isa obvious that all probe droplets presented a spherical shape. The prepared surfaces exhibited hydrophobic properties for different liquids, indicating the universality of the superhydrophobic coating.

The modified surface showed a large area of SiO_2_ and ZnO aggregate with close-packed structure formed on the substrate. Hierarchical micro-nano structures and low-surface-energy coating are two indispensable factors to construct the superhydrophobic surface. It was shown that the aggregates are composed of microscale and nanoscale spherical structures. The hierarchical micro-nano structures produce enough surface roughness for generating superhydrophobicity [16]. This is because the MTMS with abundant methyl functional groups is hydrophobic molecules, which would penetrate into the spaces among SiO_2_ nanoparticles and interconnect these particles to form a low-surface coating [44].

### 3.3. Chemical Composition of Superhydrophobic Coatings

**Figure 7:**
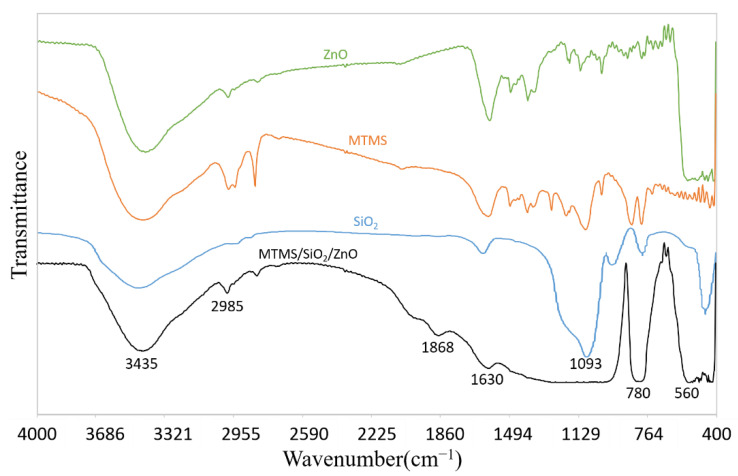
FTIR spectra of MTMS, SiO_2_, ZnO, and MTMS/SiO_2_/ZnO coating.

A small amount of powder from the surface layer of the coating was mixed and pressed with KBr powder. The ambient humidity was 63%. The infrared spectrum was tested by Fourier infrared spectrometer. FTIR spectra of the superhydrophobic film is shown in Figure 7. The O-H stretching vibration peak of intermolecular hydrogen bond is at 3435 cm^−1^. The C-H stretching vibration absorption peak is at 3000~2850 cm^−1^, indicating that the products of MTMS hydrolysis include -CH_3_. One MTMS molecule contains one methyl and three methoxy, and its surface energy is less than 25 $\mathrm{mJ}/m^{2}$ [47]. The C-H stretching vibration peak at 2985 cm^−1^ indicates that the hydrolyzed MTMS and SiO_2_ reacted to attach -CH_3_ to the surface of SiO_2._ The characteristic absorption peaks of Si-CH_3_ 780 cm^−1^ indicate that the MTMS hydrolysate and organic SiO_2_ coatings contain -CH_3_. The hydroxyl bending vibration characteristic peak of water is at 1630 cm^−1^, which may be residual adsorbed water or crystal water in the coating. It can be seen that all four contain the hydroxyl bending vibration characteristic peak of water. The peak here in the coating is significantly weaker, indicating that the hydrolytic condensation reaction of MTMS with the crystalline or adsorbed water in ZnO and SiO_2_ has occurred. Heating catalyzes the hydrolysis condensation reaction of MTMS. The vibration absorption peaks of 1000~1250 cm^−1^ are strong and wide, which is the vibration absorption band of Si-O caused by Si-O, Si-O-Si, Si-O-C, and O-Si-O. The intensity weakens and widens, indicating that MTMS cross-linked with the -OH on the surface of SiO_2_ particles and wrapped the Si-O-Si bond inside the SiO_2_ [48]. After modification by MTMS, the characteristic peak of ZnO disappeared within 455–1300 cm^−1^, and the characteristic peak of SiO_2_ disappeared at 1093 cm^−1^. It indicates that the methyl group produced by the hydrolytic condensation of MTMS successfully modified ZnO and SiO_2_. The hydrophobic group wraps the -OH inside, and the coating exhibits superhydrophobicity [47]. The analysis concluded that after modification by MTMS, its methyl group was successfully grafted onto the surface of ZnO and silica, and the -OH in the coating was mainly from MTMS with low surface energy [47]. It was shown that the hydrolyzation–condensation reaction occurs in the process of modification of SiO_2_ and ZnO by MTMS. The crosslinking reaction occurs between MTMS and -OH on the surface of SiO_2_ and ZnO particles, which envelops SiO_2_ particles and generates a Si-O-Si inorganic skeleton structure containing organic groups [46,49].

**Figure 8:**
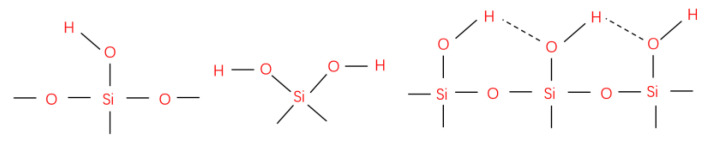
Schematic diagram of isolated silanol, geminal silanol, and vicinal silanol.

The surface of nano SiO_2_ contains -OH groups ($4.6\;\pm \;0.5)/{\mathrm{nm}}^{2}$), which provides the possibility of chemical modification. Three kinds of hydroxyl groups on the surface of SiO_2_ are shown in Figure 8, including the free hydroxyl group, twin hydroxyl group, and associated hydroxyl group [50].

The three hydrolyzed methoxyl groups of the MTMS monomer are hydrolyzed and polymerized in a three-step reaction [51], as shown in the following reaction:

1. Hydrolysis:
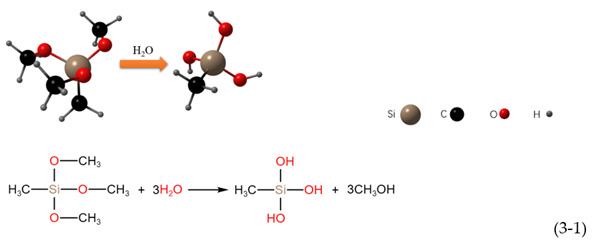
2. Water condensation:
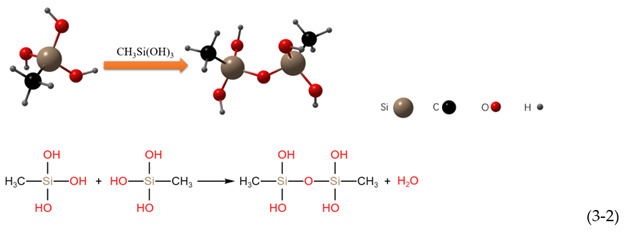
3. Alcohol condensation:
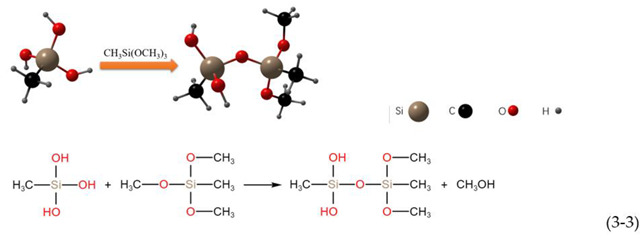

It can be seen from the above reactions that each monomer of MTMS contains one nonhydrolyzable Si-CH_3_ group. In principle, the hydroxyl group formed by the hydrolysis of methoxy react with hydroxyl groups on the surface of nano SiO_2_ and micron ZnO [51].

**Figure 9:**
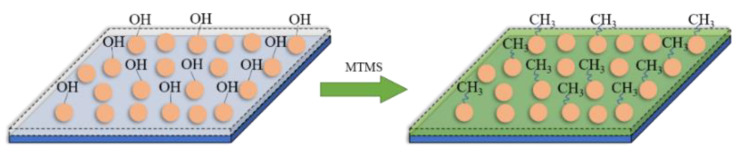
The modification of Nano SiO_2_ and micron ZnO.

The hydrolysis product of MTMS contains many -CH_3_, and the surface of nano SiO_2_ and micron ZnO contains many -OH. A highly cross-linked network polymer was formed by the above hydrolysis addition reaction at 330 °C. The -CH_3_ was successfully grafted onto the surface of the nano SiO_2_ and micron ZnO, making the coating exhibit superhydrophobicity. The modification process is shown in Figure 9; the hydrophobic methyl groups generated by the hydrolysis of MTMS replace the hydrophilic -OH on the surface of nano SiO_2_ and micron ZnO.

### 3.4. Self-Cleaning

**Figure 10:**
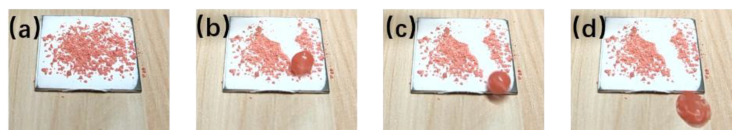
Self-cleaning process of superhydrophobic coating surface: (**a**) chalk dust was spread on the surface of the coating, (**b**) chalk dust adhering to the surface of the coating migrates to the surface of the droplets, (**c**) the water droplets roll off and carry away the chalk dust from the surface, and (**d**) the surface restored to its original clean state.

**Figure 11:**
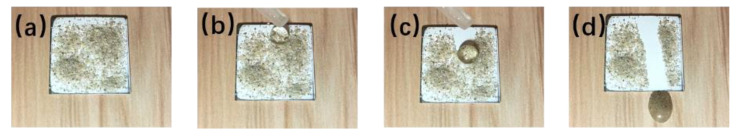
Self-cleaning process of superhydrophobic coating surface: (**a**) sand was spread on the surface of the coating, (**b**) sand adhering to the surface of the coating migrates to the surface of the droplets, (**c**) the water droplets roll off and carry away the sand from the surface, and (**d**) the surface restores to its original clean state.

**Figure 12:**
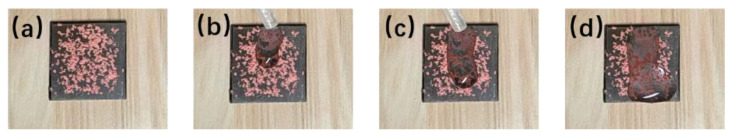
Cleaning process of Q235 steel surface: (a) chalk dust was spread on the surface of Q235 steel surface, (**b**) water drops on the surface of Q235 steel, (**c**) water flowing over the surface of Q235 steel sprinkled with chalk dust, and (**d**) large amount of chalk dust still on the surface of Q235 steel.

**Figure 13:**
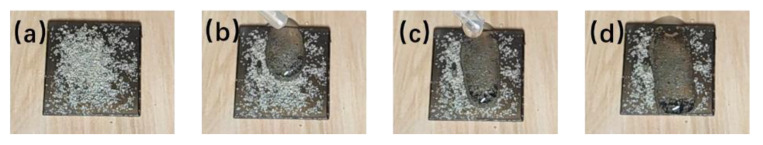
Cleaning process of Q235 steel surface: (**a**) sand was spread on the surface of Q235 steel surface, (**b**) water drops on the surface of Q235 steel, (**c**) water flowing over the surface of Q235 steel sprinkled with sand, and (**d**) large amount of sand still on the surface of Q235 steel.

The self-cleaning properties of superhydrophobic surfaces can protect them from contamination. The self-cleaning properties of the prepared superhydrophobic surfaces were investigated with two different contaminants, chalk powder with a particle size of 10 μm and sand with a particle size of 150 μm. A layer of chalk powder was spread on the superhydrophobic surface as shown in Figure 10a. A layer of sand was sprinkled on the superhydrophobic surface as shown in Figure 11a. Subsequently, the specimen surface was tilted about 10°. When a droplet (0.25 mL) was placed on the contaminated surface, it slid easily, sweeping away the contaminants in its path. The motion of the droplet appeared to be unhindered by particulate contaminants. There are two factors having played roles in this phenomenon. Firstly, the air trapped in the space around the microstructure reduces the contact area of the droplet with the superhydrophobic surface. In addition, the combined effect of the high capillary force of the water droplets and the low adhesion of particulate contaminants in this particle size range to the superhydrophobic surface facilitates the self-cleaning performance. The surface of the Q235 steel substrate is shown in Figure 12 and Figure 13. It can be seen that the water flowed down and did not carry away sand and chalk powder.

**Figure 14:**
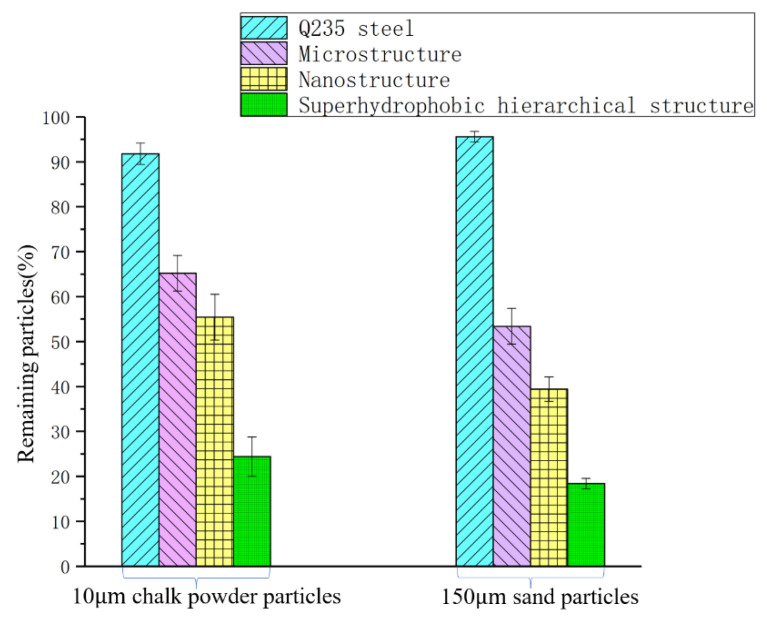
Bar chart of self-cleaning efficiency of surfaces with different wettabilities.

To further demonstrate the good self-cleaning properties of superhydrophobic surfaces, four surfaces with different wettabilities were tested. The microstructure was made of MTMS and micron-scale ZnO, and the CA was about 132.7°. The nanostructure was made of MTMS and nano SiO_2_, and the CA was about 141.5°. The superhydrophobic hierarchical structure was made of MTMS, nano SiO_2_, and micron ZnO, and the CA is about 153.9°. The water cleaning test was carried out for 1.5 min (water quantity, 10 mL) with nearly zero kinetic energy of droplets. The self-cleaning efficiencies of surfaces with different wettabilities are shown in Figure 14. Most particles (90–95%) remained on the Q235 steel surface. About 50–65% particles remained on the microstructure surface, and 35–55% particles remained on nanostructure surface. Most particles were removed from the hierarchical structured surfaces, but approximately 15–25% of particles remained. This result is generally consistent with the previously reported literature [52]. The analysis results show that the self-cleaning efficiency increases with an increasing surface CA.

### 3.5. Stability

#### 3.5.1. Atmospheric Exposure Test

**Figure 15:**
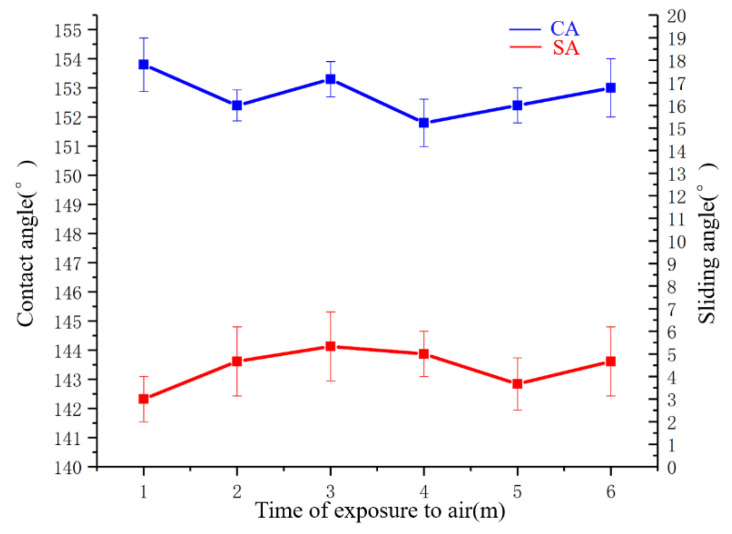
CA and SA of coatings exposed to air at different time.

Superhydrophobic coating was exposed to air in the laboratory at 27 °C room temperature, 63% humidity, and without UV light exposure. Five samples were tested. Three random locations of each coating were tested at room temperature on the coating surface. The coatings were exposed to air for six months, and CAs were performed every month. The results were shown in Figure 15.

The change of SA is shown in Figure 15. It varied between 3.0° and 4.7°. The change of CA is shown in Figure 15. The CA of the superhydrophobic coating did not change significantly with exposure to air for six months, indicating that the stability is good.

#### 3.5.2. Heat Stability

**Figure 16:**
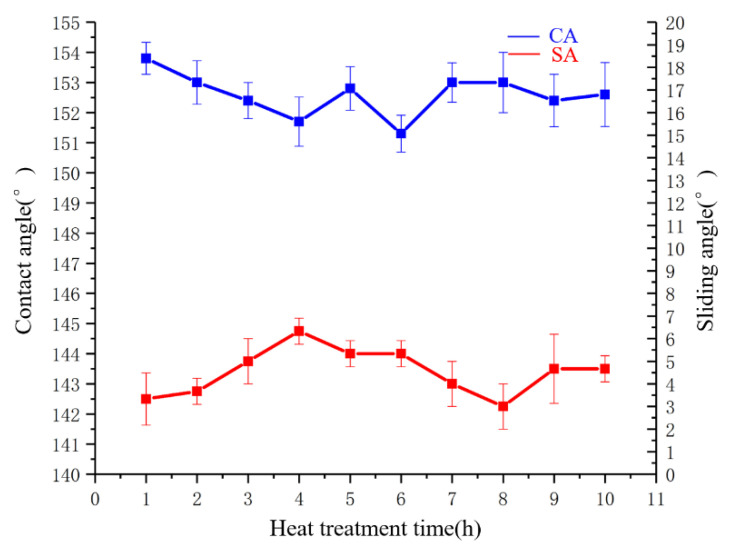
CA and SA corresponding to different treatment durations at 300 °C.

The superhydrophobic coating was placed in a crucible and then placed in a chamber furnace. Five samples were tested. Three randomly locations of each coating were tested at room temperature on the coating surface. The coatings were heated at 300 °C in the heating oven for different times, and then CA was tested. The results are shown in Figure 16.

The SA varied between 3.3° and 6.3° with no significant change. The smallest CA was 151.3° with an SA of 6.3°. The CA and SA corresponding to different treatment durations at 300 °C are shown in Figure 16.

**Figure 17:**
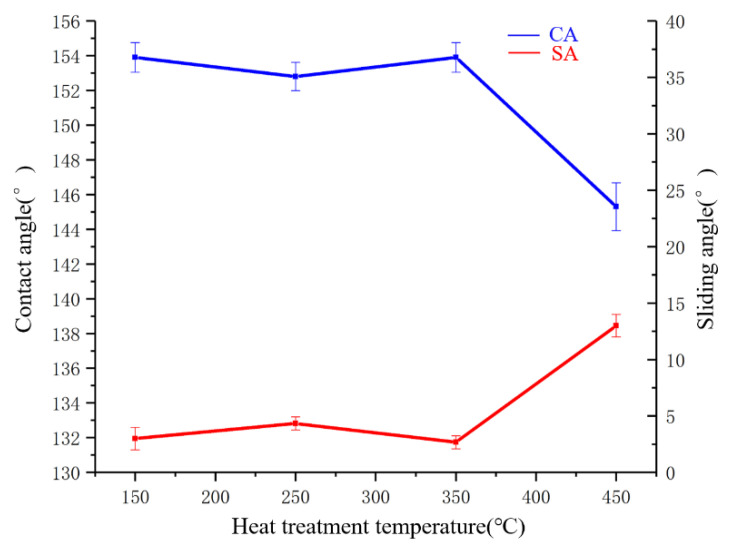
CA and SA at different heating temperature conditions.

Five samples were tested. The coatings were heated at 150 °C, 250 °C, 350 °C, and 450 °C for 30 min, respectively. After cooling, the surface CA was tested, and the results are shown in Figure 17. After two sets of heat stability tests, all specimens had a flat surface, no cracks, and no significant changes compared with those before heating.

The CA and SA at different heating temperature conditions are shown in Figure 17. The CA of the coatings did not change significantly with the increase of the heat treatment temperature from 150 °C to 350 °C. When the temperature was 450 °C, the CA decreased significantly. At the time, the CA was 145.4 °, and the SA was the largest with the value 13.0°.

**Figure 18:**
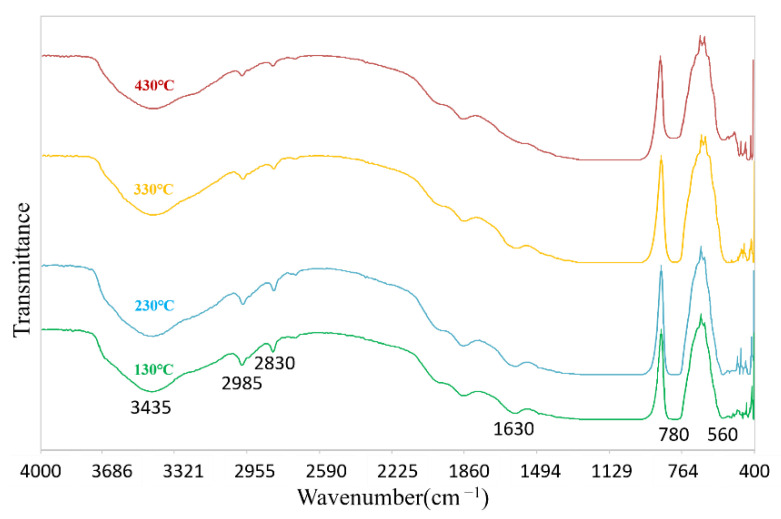
Infrared spectra of coatings after heat treatment at different temperatures.

The infrared spectra of coatings after heat treatment at different temperatures are shown in Figure 18. The broad absorption peak at 3435 cm^−1^ is the O-H stretching vibration peak; 1630 cm^−1^ is the hydroxyl bending vibration peak of water. The absorption peaks at 3435 cm^−1^ and 1630 cm^−1^ gradually weakened with the increase of temperature, and the hydroxyl bending vibration peak of water almost disappeared when the temperature increased to 430 °C, indicating that the O-H bond in the modified gum gradually disappeared with the increase of temperature [47]. The intensity of the characteristic Si-CH_3_ absorption peak at 780 cm^−1^ was essentially constant in the range of 130~330 °C, and decreased significantly at 430 °C. This may be due to the gradual decomposition of hydrophobic -CH_3_ in the coating above 400 °C, which causes the decrease of hydrophobicity and the decrease of CA. Compared with the room temperature coating, the heating at 130 °C indicates that the hydrolytic condensation reaction of MTMS was catalyzed by heating at 130 °C. From 130–330 °C, the -OH peak gradually became weaker, and the contact angle of the coating gradually increased [47].

### 3.6. Binding and Impact Test

**Figure 19:**
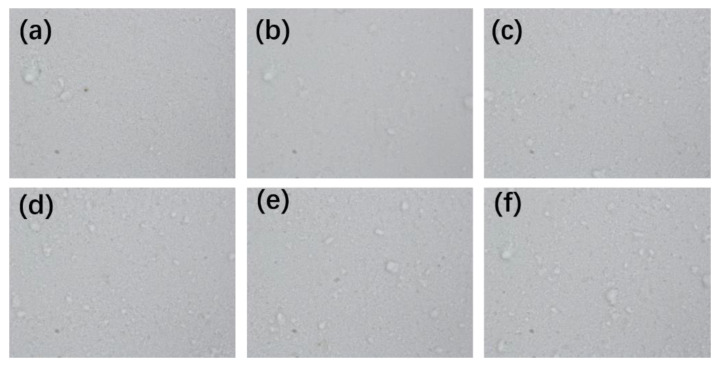
Microscopic image of superhydrophobic coating after six tape peeling. (**a**) First tape peeling, (**b**) second tape peeling, (**c**) the third time of tape peeling, (**d**) the fourth time of tape peeling, (**e**) the fifth time of tape peeling, and (**f**) the sixth time of tape peeling.

**Figure 20:**
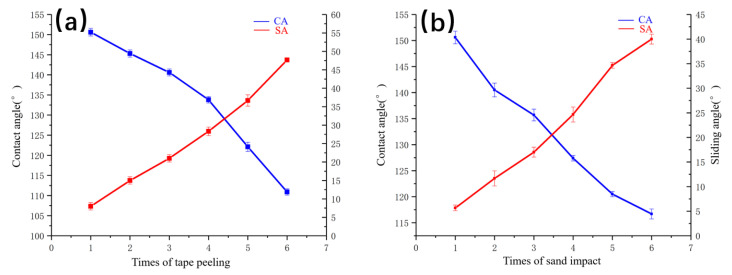
(**a**) CA and SA after tape peeling and (**b**) CA and SA after sand impact.

Five samples were used in the tape peeling test and sand impact test separately. Three random locations of each coating were tested at room temperature on the coating surface. Microscopic images of superhydrophobic coating after six strips of tape were shown in Figure 19. There was a little white powder stuck on the tape after the first tape peeling test. Five more tape peel tests were then conducted, with more white powder on the tape after each test. After six tests, the surface of the superhydrophobic coating became uneven with small pits. The CA and SA after tape peeling are shown in Figure 20; the CA reduced from 153.9° to 110.9°, and the SA increased from 3° to 47.7°.

A sand impact test was conducted, the coating was placed at an inclination of 45°, and 20 g of sand was dumped every 60 s from 30 cm above. As the number of tests with sand impacting the surface increased, the superhydrophobic surface gradually became uneven, with small pits and white powder coming off. The CA and SA after tape sand impact are shown in Figure 20. the CA reduced from 153.9° to 116.7°, and the SA increased from 3° to 40.0°.

The results of the above tests showed that the prepared bionic superhydrophobic coatings have good static stability and poor dynamic mechanical stability. This was because that the hydrolysis product of MTMS contained a large amount of -CH_3_, and a large amount of -OH on the surface of nano SiO_2_ and micron ZnO provided the possibility of chemical modification. The three formed a highly cross-linked network polymer by the above hydrolysis condensation reaction at 330 °C, resulting in the micro-nano structures. The surface of superhydrophobic surface micron-sized ZnO was covered with hydrophobic nanoscale SiO_2_ particles modified by MTMS, and the clusters adhere to each other to form a compact and unbreakable microstructure. The special structure of the aforementioned superhydrophobic surface ensures its good static stability. However, when the force of tape peeling and sand impact exceeded the force of the formed mesh, the coating surface microstructure was gradually destroyed. The wetting state of the coating surface changes from Cassie–Baxter to Wenzel [46].

### 3.7. UV Irradiation Test

**Figure 21:**
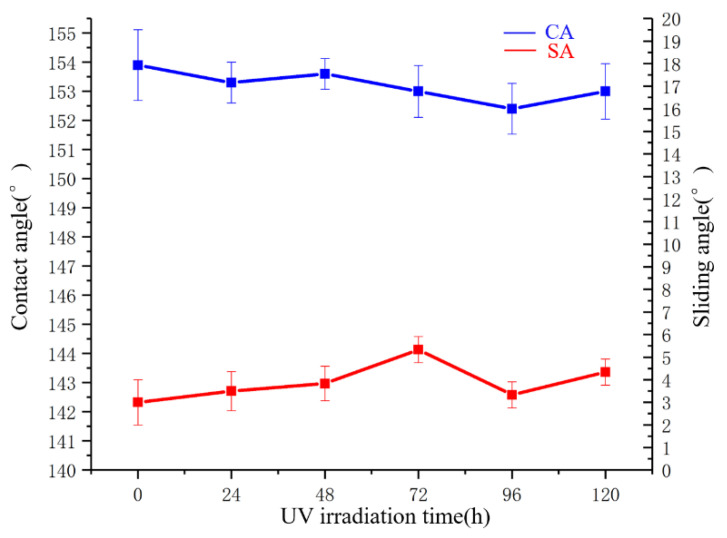
CA and SA under different UV irradiation times.

Five samples were tested. Three randomly locations of each coating were tested at room temperature on the coating surface. UV irradiation tests were conducted on the superhydrophobic coating. The CA evolution of superhydrophobic coating upon UV light irradiation is shown in Figure 21. It indicates that CA was kept almost unchanged basically upon UV light irradiation. Additionally, it could still reach as high as 153.0° and above, even though UV irradiation time extended to 120 h. The SA varied between 3.0° and 5.3°. This was because UV light could be easily absorbed by SiO_2_ nanoparticles [53]. Consequently, the superhydrophobic coating presented excellent UV resistance.

### 3.8. Icing Delay

It was shown that superhydrophobic surfaces have an icing-delay ability due to their special structure and properties, which are of great importance for practical applications [54,55,56]. The freezing of liquids on superhydrophobic surfaces is a complex process. The anti-icing ability of superhydrophobic coatings not only depends on their excellent water repellency, but also has relation with the nucleation ability of water drops on the surface [39]. The special micro and nano rough structure and low surface energy of the superhydrophobic surface reduces the adhesion of the liquid to the surface. The heterogeneous nucleation of ice is difficult on a low-energy surface; ice has a low contact area with the surface that results in a low adhesion force [16].

**Figure 22:**
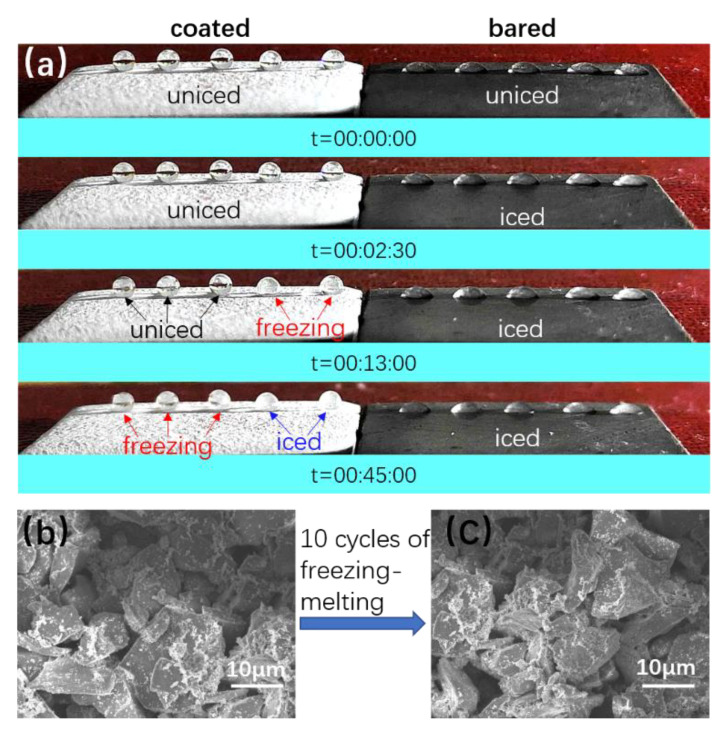
(**a**) The freezing process of a water droplet on superhydrophobic coating and Q235 steel. (**b,c**) SEM photographs of the coating after 10 freezing/melting cycles.

Three superhydrophobic coatings were tested with five water droplets on each coating. The water droplet diameter was 1.25 mm, and the test temperature was −16 °C. The rate of droplet cooling from room temperature to freezing temperature was 2 °C /h. The freezing time of static water droplets on the coating is usually used to characterize the anti-icing ability. The freezing process of water drops on the superhydrophobic coating and Q235 steel is shown in Figure 22a, the water freezing time on the bare Q235 steel and superhydrophobic coating were recorded and compared. On bare Q235 steel, the droplet was fully frozen after 2.5 min. However, on the superhydrophobic coating, it was freezing at the 13th minute. At the 45th minute, only two of the five drops were frozen. The time for a water droplet to freeze on the surface of the superhydrophobic coating is 18 times of the bare Q235 steel. The error in the time required for the first droplet to be frozen in the icing delay experiments for the three coatings was within ±0.5 min. The superhydrophobic coating showed excellent icing-delay ability. The relevant explanation for this phenomenon is as follows: (1) the contact area of water droplet with superhydrophobic surface is smaller, which provides much less area for ice nucleation, and the nucleation of water near the interface is disturbed, and (2) the air layer between water and the superhydrophobic coating hinders the heat transfer [16,39]. The Boinovich group has also studied the icing-delay properties of superhydrophobic coatings, including one study in which only two of five drops crystallized after 155 min on a superhydrophobic surface [57,58]. Compared with their prepared coatings, the superhydrophobic coatings prepared in this paper need to be improved.

Cyclic icing/melting experiment was assessed to study the lifespan of the superhydrophobic coating. After 10 icing/melting cycles, the water CA was still above 150°, and it can be found that there was no observable change on the coating surface according to the comparison of SEM images (Figure 22b,c).

## 4. Conclusions

The superhydrophobic coating with a CA of 153.9° and an SA of about 3.0° was prepared on Q235 steel substrate with 5 mL MTMS, 0.8 g of SiO_2_ nanoparticles and 0.2 g micron-grade ZnO heating 30 min at 330 °C by a simple timesaving sol-gel method. The preparation of superhydrophobic coatings uses only three reagents, which is resource-saving. The superhydrophobic coating surface was composed of rough micro/nano hierarchical structures. The structure was generated by the hydrolytic condensation cross-linking reaction of the MTMS hydrolysis products with the -hydroxyl groups on the surface of the SiO_2_ and ZnO. The superhydrophobic surface exhibited a good self-cleaning effect on both 150 μm fine sand and 10 μm chalk powder. Moreover, the superhydrophobic surface has good static stability at atmospheric exposure test and thermal stability testing. The CA of the superhydrophobic coating was still as high as 153° with exposure to air for six months. The contact angle was still as high as 151.3° after the coating was heated at 300 °C for 11 h. In addition, the superhydrophobic surface showed a self-cleaning property due to a stable gas–liquid interface provided by the micro-nano hierarchical rough structures. In addition, the superhydrophobic coating has good resistance to UV radiation and delayed icing properties. It could still reach as high as 153.0° and above, even though UV irradiation time extended to 120 h. The time for a water droplet to freeze on the surface of the superhydrophobic coating was 18 times that of the bare Q235 steel. However, in sand impact test and tape peeling test, the surface of the superhydrophobic coating became uneven with small pits. The CA after tape peeling reduced from 153.9° to 110.9°, and the SA increased from 3° to 47.7°. The CA and SA after tape sand impact reduced from 153.9° to 116.7°, and the SA increased from 3° to 40.0°. Highly cross-linked mesh structures formed gave the surface a certain static stability. When the force of tape peeling and sand impact exceeded the force of the formed mesh, the coating surface microstructure was gradually destroyed. In this work, an efficient, eco-friendly, and cost-effective sol-gel method was developed for preparing superhydrophobic surfaces. However, the prepared bionic superhydrophobic coatings have good static stability and poor dynamic mechanical stability. The improvement of mechanical stability will become the focus of subsequent research.

## Data Availability

Not applicable.
